# *cis*-{1-Butyl-3-[2-(phenyl­sulfan­yl)eth­yl]-4-imidazolin-2-yl-κ^2^ *C* ^2^,*S*′}di­chlorido­platinum(II)

**DOI:** 10.1107/S2414314620014339

**Published:** 2020-11-03

**Authors:** Bing-Bing Liang, Hong-Gang Xiong, Wan-Yu Hong, Hua-Gang Yao

**Affiliations:** a School of Chemistry and Chemical Engineering, Guangdong Pharmaceutical University, Guangdong 528458, People’s Republic of China; Okayama University, Japan

**Keywords:** crystal structure, Pt complex, *N*-heterocyclic carbene(NHC)-thio­ether ligand

## Abstract

The asymmetric unit of the title compound, [PtCl_2_(C_15_H_20_N_2_S)], comprises one Pt^II^ ion, one *N*-heterocyclic carbene(NHC)-thio­ether ligand and two chloride ions. The Pt^II^ ion is four-coordinated by one C atom and one S atom of the NHC-thio­ether ligand, and by two chloride ions, forming an approximately square-planar geometry. In the crystal, the mol­ecules are linked *via* C—H⋯Cl and C—H⋯π inter­actions, forming a layer parallel to the *ab* plane.

## Abstract 2

The title compound, [PtCl_2_(C_15_H_20_N_2_S)], was synthesized from the reaction between *N*-heterocyclic carbene(NHC)-thio­ether ligand and potassium tetra­chloro­platinate. In the crystal, the mol­ecules are linked *via* C—H⋯Cl and C—H⋯π inter­actions, forming a layer parallel to the *ab* plane.

## Graphical abstract

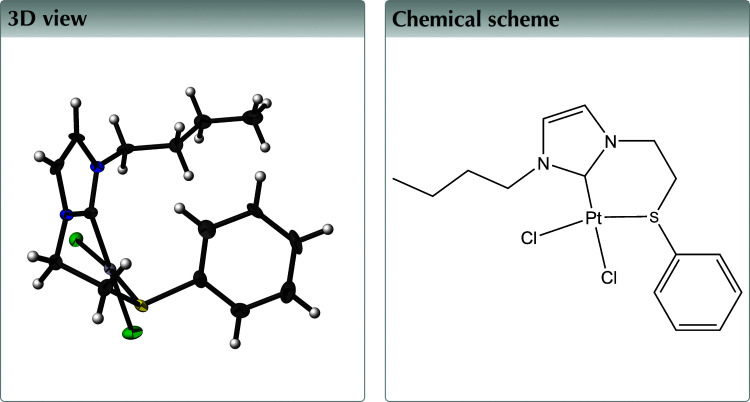

## Structure description

Nitro­gen heterocyclic carbene (NHC) exhibits attractive advantages such as simple operation and mild conditions in organic catalytic synthesis (Enders *et al.*, 2007 ▸). In addition, as a neutral two-electron donor, NHC is currently regarded as the most effective ligand for the synthesis of new organometallic complexes owing to its unique features (Hahn & Jahnke, 2008 ▸; Nelson & Nolan, 2013 ▸). The first distinctive characteristic is the strong donor property of NHC ligands, which makes the inter­action with metal center closer (Perrin *et al.*, 2001 ▸; Chianese *et al.*, 2003 ▸). The second one is that NHC can be flexibly modified by introducing functional groups onto the nitro­gen atoms of the *N*-heterocycle ring. Over the past two decades, numerous attempts have been made to construct diverse donor-functionalized NHCs and their organometallic complexes, and N-, O- and P-functionalized NHCs have been developed and applied in organic synthesis, drug discovery and materials science (Kühl, 2007 ▸). However, there are still rare investigations of NHC with S-donor complexes (Liu *et al.*, 2017 ▸). As soft and electron-rich ligands, thio­ethers usually have versatile coordination chemistry, and can form strong *M*—S bonds with the metal center (Bierenstiel & Cross, 2011 ▸; Yuan & Huynh, 2012 ▸). The development of new organometallic complexes bearing NHC-thio­ether ligands (Rosen *et al.*, 2013 ▸) is thus highly desirable. In recent years, NHC complexes with group 10 metals have received increasing attention because of their catalytic activities. In contrast to complexes of lighter homologues, Pt^II^-NHC complexes have been less well studied. The novel title metal Pt^II^ complex combined with an NHC-thio­ether ligand was designed and synthesized.

**Figure 1:**
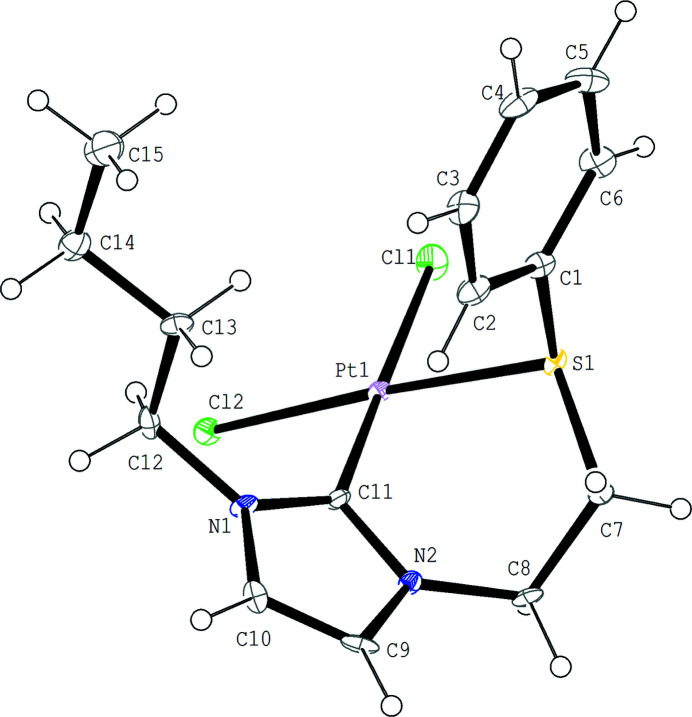
The structure of the title complex, with the atom-labelling scheme. Displacement ellipsoids are drawn at the 30% probability level.

**Figure 2:**
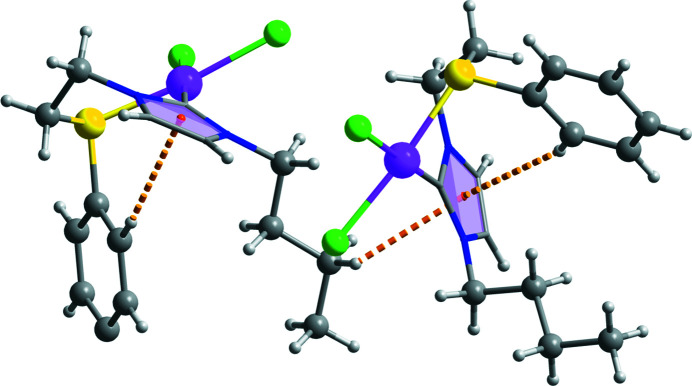
A packing diagram of the title compound, showing intra- and inter­molecular C—H⋯π inter­actions (dashed lines).

The asymmetric unit of the title complex is composed of one Pt^II^ ion, one NHC-thio­ether ligand, and two chloride ions. As shown in Fig. 1 ▸, the Pt^II^ ion is four-coordinated by one C atom and one S atom of the NHC-thio­ether ligand, and by two chloride ions in a nearly square-planar environment. The thio­ether side chain coordinates to the Pt^II^ atom in a chelating fashion, forming a six-membered ring with a distorted boat conformation. The Pt—C and Pt—S bond lengths are 1.968 (12) and 2.266 (3) Å, respectively, while the C—Pt—S bond angle is 87.93 (11)°. The two Pt—Cl bond lengths are different from each other [Pt1—Cl1 = 2.360 (3) Å and Pt1—Cl2 = 2.329 (3) Å]. In the crystal, mol­ecules are linked *via* C—H⋯Cl and C—H⋯π inter­actions (Table 1 ▸), forming a layer parallel to the *ab* plane (Figs. 2 ▸ and 3 ▸). A weak intra­molecular C—H⋯π inter­action is also observed.

## Synthesis and crystallization

*N*-Heterocyclic carbene (NHC)-thio­ether ligand was synthesized by a slight modification of a reported procedure (Liu *et al.*, 2017 ▸). Butyl-imidazole and 2-chloro­ethyl­benzene sulfide (molar ratio 1: 1) were dissolved in aceto­nitrile at 393 K for 2 days to obtain a dark-brown liquid, and then the solvent was removed by evaporation. The residue was washed repeatedly with diethyl ether, and a brownish-yellow solid was obtained.

The title complex was synthesized from the reaction of the NHC-thio­ether ligand with potassium tetra­chloro­platinate. A reaction tube was charged with the NHC-thio­ether ligand (0.1710 g, 0.576 m*M*) and 6 ml of aceto­nitrile. The tube was evacuated and back-filled with nitro­gen. Then a solution of potassium tetra­chloro­platinate (0.200 g, 0.480 m*M*) in 2 ml of water was added in the dark. Keeping it in the dark, the reaction mixture was allowed to stir at 353 K for 24 h. The mixture was concentrated *in vacuo* and purified by silica gel column chromatography. Pale-yellow rectangular crystals were obtained from the solution at room temperature.

## Refinement

Crystal data, data collection and structure refinement details are summarized in Table 2 ▸. The anisotropy of displacement ellipsoid of atom C9 was restrained with *ISOR*.

## Supplementary Material

Crystal structure: contains datablock(s) global, I. DOI: 10.1107/S2414314620014339/is5540sup1.cif

Structure factors: contains datablock(s) I. DOI: 10.1107/S2414314620014339/is5540Isup2.hkl

CCDC reference: 2041081

Additional supporting information:  crystallographic information; 3D view; checkCIF report

## Figures and Tables

**Figure 3:**
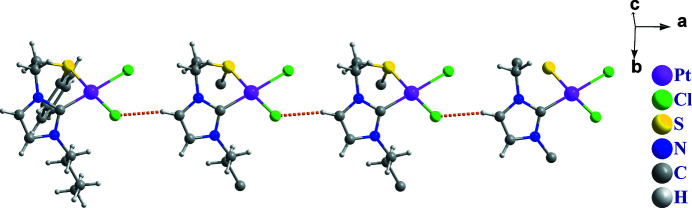
A view of the crystal packing of the title complex. Dashed lines denote the inter­molecular C—H⋯Cl hydrogen bonds.

**Table 1:** Hydrogen-bond geometry (Å, °) *Cg*1 is the centroid of the N1/C10/C9/N2/C11 ring.

*D*—H⋯*A*	*D*—H	H⋯*A*	*D*⋯*A*	*D*—H⋯*A*
C9—H9⋯Cl2^i^	0.93	2.58	3.485 (12)	163
C2—H2⋯*Cg*1	0.93	2.98	3.828 (13)	151
C14—H14*A*⋯*Cg*1^ii^	0.97	2.82	3.480 (13)	126

Symmetry codes: (i) 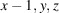 ; (ii) 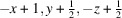 .

**Table 2:** Experimental details

Crystal data	
Chemical formula	[PtCl_2_(C_15_H_20_N_2_S)]
*M*_r_	526.38
Crystal system, space group	Orthorhombic, *P*2_1_2_1_2_1_
Temperature (K)	100
*a*, *b*, *c* (Å)	8.4254 (3), 10.1535 (4), 20.2262 (10)
*V* (Å^3^)	1730.30 (13)
*Z*	4
Radiation type	Mo *K*α
μ (mm^−1^)	8.53
Crystal size (mm)	0.12 × 0.11 × 0.09

Data collection	
Diffractometer	Rigaku Oxford Diffraction SuperNova, Dual, Cu at zero, AtlasS2
Absorption correction	Multi-scan (*CrysAlis PRO*; Rigaku OD, 2015 ▸)
*T*_min_, *T*_max_	0.310, 1.000
No. of measured, independent and observed [*I* > 2σ(*I*)] reflections	11246, 3045, 2911
*R*_int_	0.050
(sin θ/λ)_max_ (Å^−1^)	0.595

Refinement	
*R*[*F* ^2^ > 2σ(*F* ^2^)], *wR*(*F* ^2^), *S*	0.033, 0.073, 1.10
No. of reflections	3045
No. of parameters	190
No. of restraints	6
H-atom treatment	H-atom parameters constrained
Δρ_max_, Δρ_min_ (e Å^−3^)	1.27, −0.90
Absolute structure	Flack *x* determined using 1166 quotients [(*I* ^+^)−(*I* ^−^)]/[(*I* ^+^)+(*I* ^−^)] (Parsons *et al.*, 2013 ▸)
Absolute structure parameter	−0.020 (7)

Computer programs: *CrysAlis PRO* (Rigaku OD, 2015 ▸), *SHELXT* (Sheldrick, 2015*a* ▸), *SHELXL2018/3* (Sheldrick, 2015*b* ▸) and *OLEX2* (Dolomanov *et al.*, 2009 ▸).

## full crystallographic data

### Crystal data

**Table:**

[PtCl_2_(C_15_H_20_N_2_S)]	*D*_x_ = 2.021 Mg m^−^^3^
*M**_r_* = 526.38	Mo *K*α radiation, λ = 0.71073 Å
Orthorhombic, *P*2_1_2_1_2_1_	Cell parameters from 6072 reflections
*a* = 8.4254 (3) Å	θ = 2.3–29.3°
*b* = 10.1535 (4) Å	µ = 8.53 mm^−^^1^
*c* = 20.2262 (10) Å	*T* = 100 K
*V* = 1730.30 (13) Å^3^	Block, colourless
*Z* = 4	0.12 × 0.11 × 0.09 mm
*F*(000) = 1008	

### Data collection

**Table:**

Rigaku Oxford Diffraction SuperNova, Dual, Cu at zero, AtlasS2 diffractometer	3045 independent reflections
Radiation source: micro-focus sealed X-ray tube, SuperNova (Mo) X-ray Source	2911 reflections with *I* > 2σ(*I*)
Mirror monochromator	*R*_int_ = 0.050
Detector resolution: 5.2684 pixels mm^-1^	θ_max_ = 25.0°, θ_min_ = 2.0°
ω scans	*h* = −9→10
Absorption correction: multi-scan (CrysAlisPro; Rigaku OD, 2015)	*k* = −10→12
*T*_min_ = 0.310, *T*_max_ = 1.000	*l* = −24→22
11246 measured reflections	

### Refinement

**Table:**

Refinement on *F*^2^	Hydrogen site location: inferred from neighbouring sites
Least-squares matrix: full	H-atom parameters constrained
*R*[*F*^2^ > 2σ(*F*^2^)] = 0.033	*w* = 1/[σ^2^(*F*_o_^2^) + (0.0237*P*)^2^ + 8.5737*P*] where *P* = (*F*_o_^2^ + 2*F*_c_^2^)/3
*wR*(*F*^2^) = 0.073	(Δ/σ)_max_ < 0.001
*S* = 1.10	Δρ_max_ = 1.27 e Å^−^^3^
3045 reflections	Δρ_min_ = −0.90 e Å^−^^3^
190 parameters	Absolute structure: Flack *x* determined using 1166 quotients [(*I*^+^)-(*I*^-^)]/[(*I*^+^)+(*I*^-^)] (Parsons *et al.*, 2013)
6 restraints	Absolute structure parameter: −0.020 (7)
Primary atom site location: dual	

### Special details

**Table:**

Geometry. All esds (except the esd in the dihedral angle between two l.s. planes) are estimated using the full covariance matrix. The cell esds are taken into account individually in the estimation of esds in distances, angles and torsion angles; correlations between esds in cell parameters are only used when they are defined by crystal symmetry. An approximate (isotropic) treatment of cell esds is used for estimating esds involving l.s. planes.

### Fractional atomic coordinates and isotropic or equivalent isotropic displacement parameters (Å^2^)

**Table:**

	*x*	*y*	*z*	*U*_iso_*/*U*_eq_	
Pt1	0.63221 (5)	0.45155 (4)	0.36816 (2)	0.01172 (13)	
Cl1	0.8671 (4)	0.3701 (3)	0.41687 (15)	0.0251 (7)	
Cl2	0.7685 (3)	0.4970 (3)	0.27062 (15)	0.0166 (7)	
S1	0.5012 (4)	0.3876 (3)	0.46058 (16)	0.0132 (6)	
N1	0.3999 (10)	0.6337 (9)	0.2997 (5)	0.013 (2)	
N2	0.2959 (10)	0.4495 (10)	0.3301 (5)	0.013 (2)	
C1	0.4671 (13)	0.5293 (12)	0.5109 (6)	0.017 (3)	
C2	0.3753 (16)	0.6352 (11)	0.4903 (6)	0.022 (3)	
H2	0.324679	0.633418	0.449406	0.027*	
C3	0.3602 (18)	0.7440 (11)	0.5317 (6)	0.023 (3)	
H3	0.301393	0.816485	0.517916	0.027*	
C4	0.4319 (15)	0.7454 (13)	0.5932 (7)	0.026 (3)	
H4	0.421705	0.818696	0.620501	0.031*	
C5	0.5187 (15)	0.6376 (14)	0.6141 (7)	0.028 (3)	
H5	0.565556	0.637828	0.655695	0.034*	
C6	0.5362 (14)	0.5289 (13)	0.5730 (6)	0.023 (3)	
H6	0.594069	0.456209	0.587208	0.028*	
C7	0.2978 (14)	0.3408 (13)	0.4385 (6)	0.017 (3)	
H7A	0.270431	0.259251	0.460755	0.021*	
H7B	0.225027	0.408416	0.453583	0.021*	
C8	0.2785 (13)	0.3224 (11)	0.3637 (7)	0.016 (3)	
H8A	0.358118	0.261421	0.347493	0.019*	
H8B	0.174644	0.285757	0.354193	0.019*	
C9	0.1750 (14)	0.5213 (13)	0.3000 (6)	0.020 (3)	
H9	0.069889	0.495503	0.294379	0.024*	
C10	0.2414 (14)	0.6358 (12)	0.2805 (6)	0.015 (3)	
H10	0.190345	0.703945	0.258351	0.018*	
C11	0.4343 (14)	0.5184 (11)	0.3288 (6)	0.016 (3)	
C12	0.5054 (15)	0.7492 (12)	0.2959 (6)	0.018 (3)	
H12A	0.477671	0.801717	0.257510	0.021*	
H12B	0.614370	0.719995	0.290687	0.021*	
C13	0.4914 (14)	0.8325 (11)	0.3576 (6)	0.018 (3)	
H13A	0.533278	0.783493	0.394901	0.022*	
H13B	0.380163	0.850067	0.366229	0.022*	
C14	0.5803 (14)	0.9636 (13)	0.3520 (6)	0.023 (3)	
H14A	0.691832	0.946477	0.343681	0.028*	
H14B	0.538795	1.012962	0.314704	0.028*	
C15	0.5640 (17)	1.0449 (15)	0.4139 (7)	0.037 (4)	
H15A	0.620925	1.126163	0.408726	0.055*	
H15B	0.453847	1.063416	0.421815	0.055*	
H15C	0.606756	0.996986	0.450767	0.055*	

### Atomic displacement parameters (Å^2^)

**Table:**

	*U*^11^	*U*^22^	*U*^33^	*U*^12^	*U*^13^	*U*^23^
Pt1	0.0068 (2)	0.0125 (2)	0.0158 (2)	−0.00009 (17)	0.0003 (2)	−0.0010 (2)
Cl1	0.0119 (14)	0.0317 (17)	0.0317 (18)	0.0032 (16)	−0.0018 (16)	0.0097 (14)
Cl2	0.0094 (15)	0.0230 (15)	0.0174 (16)	−0.0007 (11)	0.0022 (12)	−0.0003 (12)
S1	0.0116 (15)	0.0097 (15)	0.0183 (17)	−0.0005 (11)	0.0018 (13)	0.0005 (13)
N1	0.004 (5)	0.014 (5)	0.022 (6)	0.001 (4)	−0.004 (4)	0.000 (4)
N2	0.010 (3)	0.012 (3)	0.016 (3)	0.000 (2)	−0.001 (2)	0.001 (2)
C1	0.011 (6)	0.020 (7)	0.020 (7)	0.000 (5)	0.004 (5)	−0.006 (6)
C2	0.013 (6)	0.024 (7)	0.030 (7)	−0.002 (6)	0.002 (7)	−0.003 (6)
C3	0.029 (7)	0.010 (6)	0.029 (8)	0.005 (6)	0.002 (7)	−0.003 (5)
C4	0.021 (7)	0.023 (7)	0.033 (9)	−0.006 (5)	0.011 (6)	−0.018 (7)
C5	0.017 (7)	0.042 (9)	0.026 (9)	0.001 (6)	−0.005 (6)	−0.012 (7)
C6	0.013 (7)	0.025 (8)	0.032 (8)	0.007 (5)	−0.004 (5)	0.000 (6)
C7	0.015 (6)	0.022 (7)	0.016 (7)	−0.007 (5)	−0.002 (5)	0.001 (6)
C8	0.008 (6)	0.016 (6)	0.023 (7)	−0.003 (4)	−0.005 (6)	−0.004 (6)
C9	0.012 (7)	0.031 (8)	0.018 (7)	0.005 (5)	−0.012 (5)	−0.005 (6)
C10	0.015 (6)	0.013 (6)	0.017 (7)	0.010 (5)	0.003 (5)	0.004 (6)
C11	0.014 (6)	0.012 (7)	0.020 (7)	−0.005 (5)	−0.007 (5)	0.003 (5)
C12	0.013 (7)	0.022 (7)	0.018 (7)	0.003 (5)	0.002 (5)	0.010 (6)
C13	0.010 (6)	0.021 (6)	0.023 (8)	0.000 (5)	−0.005 (5)	−0.003 (6)
C14	0.016 (6)	0.022 (7)	0.031 (8)	0.000 (5)	−0.003 (5)	0.003 (6)
C15	0.039 (8)	0.024 (7)	0.047 (9)	−0.001 (7)	−0.017 (7)	0.007 (8)

### Geometric parameters (Å, º)

**Table:**

Pt1—Cl1	2.360 (3)	C6—H6	0.9300
Pt1—Cl2	2.329 (3)	C7—H7A	0.9700
Pt1—S1	2.266 (3)	C7—H7B	0.9700
Pt1—C11	1.968 (12)	C7—C8	1.533 (18)
S1—C1	1.786 (12)	C8—H8A	0.9700
S1—C7	1.834 (12)	C8—H8B	0.9700
N1—C10	1.390 (15)	C9—H9	0.9300
N1—C11	1.343 (15)	C9—C10	1.349 (17)
N1—C12	1.474 (15)	C10—H10	0.9300
N2—C8	1.465 (15)	C12—H12A	0.9700
N2—C9	1.393 (14)	C12—H12B	0.9700
N2—C11	1.360 (14)	C12—C13	1.513 (17)
C1—C2	1.388 (17)	C13—H13A	0.9700
C1—C6	1.385 (17)	C13—H13B	0.9700
C2—H2	0.9300	C13—C14	1.532 (16)
C2—C3	1.394 (16)	C14—H14A	0.9700
C3—H3	0.9300	C14—H14B	0.9700
C3—C4	1.383 (19)	C14—C15	1.507 (18)
C4—H4	0.9300	C15—H15A	0.9600
C4—C5	1.383 (19)	C15—H15B	0.9600
C5—H5	0.9300	C15—H15C	0.9600
C5—C6	1.388 (19)		
			
Cl2—Pt1—Cl1	90.54 (11)	N2—C8—C7	109.9 (10)
S1—Pt1—Cl1	87.93 (11)	N2—C8—H8A	109.7
S1—Pt1—Cl2	174.77 (11)	N2—C8—H8B	109.7
C11—Pt1—Cl1	179.0 (4)	C7—C8—H8A	109.7
C11—Pt1—Cl2	90.4 (4)	C7—C8—H8B	109.7
C11—Pt1—S1	91.1 (4)	H8A—C8—H8B	108.2
C1—S1—Pt1	108.5 (4)	N2—C9—H9	127.0
C1—S1—C7	101.4 (6)	C10—C9—N2	106.0 (10)
C7—S1—Pt1	109.2 (4)	C10—C9—H9	127.0
C10—N1—C12	123.6 (10)	N1—C10—H10	126.1
C11—N1—C10	110.1 (10)	C9—C10—N1	107.7 (10)
C11—N1—C12	125.9 (9)	C9—C10—H10	126.1
C9—N2—C8	126.2 (9)	N1—C11—Pt1	131.4 (8)
C11—N2—C8	123.2 (9)	N1—C11—N2	105.7 (9)
C11—N2—C9	110.5 (10)	N2—C11—Pt1	122.8 (8)
C2—C1—S1	122.8 (9)	N1—C12—H12A	109.5
C6—C1—S1	116.6 (10)	N1—C12—H12B	109.5
C6—C1—C2	120.7 (12)	N1—C12—C13	110.8 (10)
C1—C2—H2	120.5	H12A—C12—H12B	108.1
C1—C2—C3	118.9 (12)	C13—C12—H12A	109.5
C3—C2—H2	120.5	C13—C12—H12B	109.5
C2—C3—H3	119.7	C12—C13—H13A	109.0
C4—C3—C2	120.7 (12)	C12—C13—H13B	109.0
C4—C3—H3	119.7	C12—C13—C14	112.7 (10)
C3—C4—H4	120.1	H13A—C13—H13B	107.8
C3—C4—C5	119.8 (12)	C14—C13—H13A	109.0
C5—C4—H4	120.1	C14—C13—H13B	109.0
C4—C5—H5	119.9	C13—C14—H14A	109.3
C4—C5—C6	120.2 (13)	C13—C14—H14B	109.3
C6—C5—H5	119.9	H14A—C14—H14B	108.0
C1—C6—C5	119.7 (12)	C15—C14—C13	111.7 (11)
C1—C6—H6	120.2	C15—C14—H14A	109.3
C5—C6—H6	120.2	C15—C14—H14B	109.3
S1—C7—H7A	109.3	C14—C15—H15A	109.5
S1—C7—H7B	109.3	C14—C15—H15B	109.5
H7A—C7—H7B	107.9	C14—C15—H15C	109.5
C8—C7—S1	111.8 (8)	H15A—C15—H15B	109.5
C8—C7—H7A	109.3	H15A—C15—H15C	109.5
C8—C7—H7B	109.3	H15B—C15—H15C	109.5
			
Pt1—S1—C1—C2	62.3 (11)	C8—N2—C9—C10	175.0 (11)
Pt1—S1—C1—C6	−118.3 (9)	C8—N2—C11—Pt1	3.2 (16)
Pt1—S1—C7—C8	14.3 (10)	C8—N2—C11—N1	−174.1 (10)
S1—C1—C2—C3	−177.4 (10)	C9—N2—C8—C7	−108.8 (12)
S1—C1—C6—C5	178.0 (10)	C9—N2—C11—Pt1	178.8 (8)
S1—C7—C8—N2	−67.4 (11)	C9—N2—C11—N1	1.5 (14)
N1—C12—C13—C14	−171.7 (10)	C10—N1—C11—Pt1	−179.0 (10)
N2—C9—C10—N1	−0.9 (13)	C10—N1—C11—N2	−2.0 (14)
C1—S1—C7—C8	128.8 (9)	C10—N1—C12—C13	86.3 (13)
C1—C2—C3—C4	−2 (2)	C11—N1—C10—C9	1.8 (14)
C2—C1—C6—C5	−2.5 (19)	C11—N1—C12—C13	−85.0 (14)
C2—C3—C4—C5	0 (2)	C11—N2—C8—C7	66.0 (14)
C3—C4—C5—C6	1 (2)	C11—N2—C9—C10	−0.4 (14)
C4—C5—C6—C1	0 (2)	C12—N1—C10—C9	−170.7 (11)
C6—C1—C2—C3	3.2 (19)	C12—N1—C11—Pt1	−6.7 (19)
C7—S1—C1—C2	−52.6 (11)	C12—N1—C11—N2	170.3 (10)
C7—S1—C1—C6	126.8 (10)	C12—C13—C14—C15	179.8 (10)

### Hydrogen-bond geometry (Å, º)

*Cg*1 is the centroid of the N1/C10/C9/N2/C11 ring.

**Table:**

*D*—H···*A*	*D*—H	H···*A*	*D*···*A*	*D*—H···*A*
C9—H9···Cl2^i^	0.93	2.58	3.485 (12)	163
C2—H2···*Cg*1	0.93	2.98	3.828 (13)	151
C14—H14*A*···*Cg*1^ii^	0.97	2.82	3.480 (13)	126

Symmetry codes: (i) *x*−1, *y*, *z*; (ii) −*x*+1, *y*+1/2, −*z*+1/2.
